# Supplementary material for: Modeling glioblastoma heterogeneity as a dynamic network of cell states
Source: Mol Syst Biol. 2021 Sep 16;17(9):e10105. doi: 10.15252/msb.202010105 (PMC8444284; doi:10.15252/msb.202010105)
Supplement: Supplementary file 5 — Source Data for Figure 3 [file MSB-17-e10105-s001.zip › Figure3A_sourcedata/GSEA_3065/nosigdb_state5.GseaPreranked.1622718435914/RICHARDS_NOWAKOWSKI_SCIENCE_2017_RG_EARLY_UPREG.html]

Details for gene set RICHARDS\_NOWAKOWSKI\_SCIENCE\_2017\_RG\_EARLY\_UPREG[GSEA]

|  || Dataset | state5 |
| Phenotype | NoPhenotypeAvailable |
| Upregulated in class | na\_neg |
| GeneSet | RICHARDS\_NOWAKOWSKI\_SCIENCE\_2017\_RG\_EARLY\_UPREG |
| Enrichment Score (ES) | -0.61381483 |
| Normalized Enrichment Score (NES) | -2.4140394 |
| Nominal p-value | 0.0 |
| FDR q-value | 0.0 |
| FWER p-Value | 0.0 |
Table: GSEA Results Summary

  

Fig 1: Enrichment plot: RICHARDS\_NOWAKOWSKI\_SCIENCE\_2017\_RG\_EARLY\_UPREG      
 Profile of the Running ES Score & Positions of GeneSet Members on the Rank Ordered List

  

| PROBE | GENE SYMBOL | GENE\_TITLE | RANK IN GENE LIST | RANK METRIC SCORE | RUNNING ES | CORE ENRICHMENT || 1 | MDM4 |  |  | 21 | 0.811 | 0.0039 | No |
| 2 | HSP90B1 |  |  | 33 | 0.729 | 0.0084 | No |
| 3 | PON2 |  |  | 84 | 0.546 | 0.0069 | No |
| 4 | SEMA5A |  |  | 111 | 0.516 | 0.0079 | No |
| 5 | ITGB8 |  |  | 126 | 0.503 | 0.0102 | No |
| 6 | TTYH1 |  |  | 141 | 0.489 | 0.0125 | No |
| 7 | FAT1 |  |  | 155 | 0.478 | 0.0147 | No |
| 8 | MCAM |  |  | 174 | 0.460 | 0.0162 | No |
| 9 | SLC3A2 |  |  | 222 | 0.424 | 0.0141 | No |
| 10 | SLC25A37 |  |  | 223 | 0.422 | 0.0174 | No |
| 11 | SRPRB |  |  | 251 | 0.408 | 0.0175 | No |
| 12 | JAM3 |  |  | 276 | 0.395 | 0.0178 | No |
| 13 | HELLS |  |  | 280 | 0.393 | 0.0205 | No |
| 14 | CCNL1 |  |  | 300 | 0.382 | 0.0213 | No |
| 15 | CREB5 |  |  | 321 | 0.373 | 0.0219 | No |
| 16 | FKBP10 |  |  | 337 | 0.365 | 0.0230 | No |
| 17 | RBM39 |  |  | 353 | 0.360 | 0.0241 | No |
| 18 | SPCS1 |  |  | 375 | 0.351 | 0.0244 | No |
| 19 | TRA2A |  |  | 419 | 0.335 | 0.0221 | No |
| 20 | SLC5A3 |  |  | 440 | 0.327 | 0.0223 | No |
| 21 | ABHD3 |  |  | 458 | 0.323 | 0.0229 | No |
| 22 | OFD1 |  |  | 502 | 0.311 | 0.0203 | No |
| 23 | RFX4 |  |  | 509 | 0.308 | 0.0220 | No |
| 24 | DNAJC1 |  |  | 517 | 0.306 | 0.0236 | No |
| 25 | BRCA1 |  |  | 522 | 0.305 | 0.0255 | No |
| 26 | IGF2BP2 |  |  | 524 | 0.305 | 0.0278 | No |
| 27 | AGO3 |  |  | 532 | 0.302 | 0.0294 | No |
| 28 | HMGA2 |  |  | 547 | 0.298 | 0.0301 | No |
| 29 | ATP1B1 |  |  | 548 | 0.298 | 0.0325 | No |
| 30 | FGFR3 |  |  | 567 | 0.293 | 0.0327 | No |
| 31 | DCLRE1C |  |  | 580 | 0.290 | 0.0336 | No |
| 32 | AHI1 |  |  | 609 | 0.283 | 0.0325 | No |
| 33 | CREB3L2 |  |  | 629 | 0.279 | 0.0325 | No |
| 34 | ARHGEF10 |  |  | 712 | 0.259 | 0.0249 | No |
| 35 | SREK1 |  |  | 729 | 0.255 | 0.0251 | No |
| 36 | SCAF11 |  |  | 872 | 0.229 | 0.0103 | No |
| 37 | WEE1 |  |  | 919 | 0.222 | 0.0067 | No |
| 38 | XPO1 |  |  | 933 | 0.220 | 0.0069 | No |
| 39 | ASPM |  |  | 980 | 0.213 | 0.0032 | No |
| 40 | TMEM97 |  |  | 995 | 0.211 | 0.0032 | No |
| 41 | CNIH1 |  |  | 1023 | 0.208 | 0.0017 | No |
| 42 | C1orf61 |  |  | 1057 | 0.204 | -0.0006 | No |
| 43 | BOC |  |  | 1113 | 0.197 | -0.0054 | No |
| 44 | PPP1R15B |  |  | 1156 | 0.191 | -0.0088 | No |
| 45 | PTAR1 |  |  | 1266 | 0.177 | -0.0202 | No |
| 46 | GPC4 |  |  | 1288 | 0.173 | -0.0213 | No |
| 47 | MYADM |  |  | 1345 | 0.167 | -0.0265 | No |
| 48 | CEP152 |  |  | 1436 | 0.159 | -0.0358 | No |
| 49 | LIFR |  |  | 1520 | 0.150 | -0.0443 | No |
| 50 | CENPF |  |  | 1531 | 0.149 | -0.0443 | No |
| 51 | TMEM170A |  |  | 1536 | 0.148 | -0.0436 | No |
| 52 | PIK3R3 |  |  | 1584 | 0.142 | -0.0480 | No |
| 53 | MTHFD2L |  |  | 1597 | 0.141 | -0.0483 | No |
| 54 | VEGFA |  |  | 1634 | 0.139 | -0.0514 | No |
| 55 | RBL1 |  |  | 1669 | 0.136 | -0.0543 | No |
| 56 | ZFHX4 |  |  | 1700 | 0.133 | -0.0567 | No |
| 57 | MIS18BP1 |  |  | 1765 | 0.129 | -0.0632 | No |
| 58 | INTS8 |  |  | 1768 | 0.128 | -0.0624 | No |
| 59 | DYRK1A |  |  | 1812 | 0.126 | -0.0665 | No |
| 60 | ZNF844 |  |  | 1895 | 0.119 | -0.0751 | No |
| 61 | TNFRSF19 |  |  | 1943 | 0.116 | -0.0797 | No |
| 62 | KIF20B |  |  | 1962 | 0.114 | -0.0809 | No |
| 63 | NOTCH3 |  |  | 1978 | 0.113 | -0.0818 | No |
| 64 | TSPAN6 |  |  | 2087 | 0.106 | -0.0936 | No |
| 65 | CEP135 |  |  | 2201 | 0.098 | -0.1060 | No |
| 66 | ASNS |  |  | 2215 | 0.097 | -0.1067 | No |
| 67 | HES1 |  |  | 2302 | 0.092 | -0.1161 | No |
| 68 | ADAMTS9 |  |  | 2340 | 0.089 | -0.1197 | No |
| 69 | MDK |  |  | 2458 | 0.082 | -0.1327 | No |
| 70 | ATAD2 |  |  | 2545 | 0.077 | -0.1422 | No |
| 71 | PEG3 |  |  | 2686 | 0.069 | -0.1580 | No |
| 72 | TUBE1 |  |  | 2712 | 0.067 | -0.1604 | No |
| 73 | PTBP1 |  |  | 2754 | 0.065 | -0.1647 | No |
| 74 | CCDC18 |  |  | 2814 | 0.062 | -0.1711 | No |
| 75 | RTKN2 |  |  | 2869 | 0.059 | -0.1769 | No |
| 76 | CHEK1 |  |  | 2877 | 0.059 | -0.1773 | No |
| 77 | SYNE2 |  |  | 2881 | 0.058 | -0.1772 | No |
| 78 | RNF139 |  |  | 2891 | 0.058 | -0.1778 | No |
| 79 | RAD51AP1 |  |  | 2905 | 0.057 | -0.1788 | No |
| 80 | TOPORS |  |  | 2949 | 0.055 | -0.1834 | No |
| 81 | CKAP2L |  |  | 2979 | 0.053 | -0.1864 | No |
| 82 | TCF7L1 |  |  | 3012 | 0.051 | -0.1897 | No |
| 83 | HDAC9 |  |  | 3051 | 0.049 | -0.1938 | No |
| 84 | KIAA1217 |  |  | 3072 | 0.048 | -0.1958 | No |
| 85 | FANCD2 |  |  | 3151 | 0.044 | -0.2045 | No |
| 86 | NUP155 |  |  | 3153 | 0.044 | -0.2043 | No |
| 87 | CBFB |  |  | 3166 | 0.043 | -0.2054 | No |
| 88 | NCAPG |  |  | 3194 | 0.041 | -0.2082 | No |
| 89 | CNTLN |  |  | 3231 | 0.040 | -0.2121 | No |
| 90 | TAF8 |  |  | 3265 | 0.038 | -0.2156 | No |
| 91 | IER5 |  |  | 3319 | 0.035 | -0.2216 | No |
| 92 | ZNF516 |  |  | 3341 | 0.034 | -0.2238 | No |
| 93 | BUB1B |  |  | 3355 | 0.034 | -0.2250 | No |
| 94 | TCF7L2 |  |  | 3356 | 0.033 | -0.2247 | No |
| 95 | CHORDC1 |  |  | 3360 | 0.033 | -0.2248 | No |
| 96 | SMC2 |  |  | 3374 | 0.032 | -0.2261 | No |
| 97 | FOXN2 |  |  | 3403 | 0.032 | -0.2291 | No |
| 98 | GPBP1L1 |  |  | 3451 | 0.029 | -0.2344 | No |
| 99 | NUP50 |  |  | 3457 | 0.029 | -0.2347 | No |
| 100 | HSPA4L |  |  | 3471 | 0.028 | -0.2360 | No |
| 101 | WDR36 |  |  | 3483 | 0.028 | -0.2371 | No |
| 102 | EFNB1 |  |  | 3659 | 0.022 | -0.2574 | No |
| 103 | SPC24 |  |  | 3726 | 0.019 | -0.2650 | No |
| 104 | RDH10 |  |  | 3750 | 0.018 | -0.2675 | No |
| 105 | ITGB3BP |  |  | 3830 | 0.014 | -0.2766 | No |
| 106 | DLGAP5 |  |  | 3907 | 0.011 | -0.2854 | No |
| 107 | KIF15 |  |  | 3950 | 0.009 | -0.2903 | No |
| 108 | MDM1 |  |  | 3979 | 0.008 | -0.2935 | No |
| 109 | NDC80 |  |  | 4034 | 0.006 | -0.2998 | No |
| 110 | PBX3 |  |  | 4054 | 0.005 | -0.3019 | No |
| 111 | TMEM258 |  |  | 4058 | 0.005 | -0.3022 | No |
| 112 | IMMP1L |  |  | 4163 | 0.000 | -0.3144 | No |
| 113 | ATF3 |  |  | 4225 | -0.002 | -0.3215 | No |
| 114 | MAGED2 |  |  | 4228 | -0.002 | -0.3217 | No |
| 115 | PRC1 |  |  | 4241 | -0.003 | -0.3231 | No |
| 116 | SOX2 |  |  | 4271 | -0.005 | -0.3265 | No |
| 117 | KDM3A |  |  | 4280 | -0.005 | -0.3274 | No |
| 118 | RAP2C |  |  | 4293 | -0.006 | -0.3287 | No |
| 119 | DARS |  |  | 4329 | -0.007 | -0.3328 | No |
| 120 | RC3H2 |  |  | 4341 | -0.007 | -0.3340 | No |
| 121 | GPCPD1 |  |  | 4357 | -0.008 | -0.3357 | No |
| 122 | MSI1 |  |  | 4443 | -0.012 | -0.3455 | No |
| 123 | ZNF433 |  |  | 4484 | -0.013 | -0.3501 | No |
| 124 | MCM4 |  |  | 4534 | -0.015 | -0.3557 | No |
| 125 | GLI3 |  |  | 4538 | -0.016 | -0.3559 | No |
| 126 | DUSP16 |  |  | 4557 | -0.016 | -0.3579 | No |
| 127 | SAR1A |  |  | 4562 | -0.016 | -0.3582 | No |
| 128 | KIF2C |  |  | 4621 | -0.019 | -0.3649 | No |
| 129 | RNPS1 |  |  | 4641 | -0.020 | -0.3669 | No |
| 130 | CENPK |  |  | 4675 | -0.021 | -0.3706 | No |
| 131 | REST |  |  | 4679 | -0.021 | -0.3708 | No |
| 132 | ZNF134 |  |  | 4722 | -0.023 | -0.3755 | No |
| 133 | WDR43 |  |  | 4725 | -0.023 | -0.3756 | No |
| 134 | DPM1 |  |  | 4747 | -0.024 | -0.3779 | No |
| 135 | KIF14 |  |  | 4758 | -0.024 | -0.3788 | No |
| 136 | CDK1 |  |  | 4766 | -0.025 | -0.3795 | No |
| 137 | BARD1 |  |  | 4776 | -0.025 | -0.3803 | No |
| 138 | CENPE |  |  | 4787 | -0.025 | -0.3813 | No |
| 139 | PRR11 |  |  | 4822 | -0.026 | -0.3851 | No |
| 140 | ID4 |  |  | 4830 | -0.027 | -0.3857 | No |
| 141 | NFKBIA |  |  | 4840 | -0.027 | -0.3865 | No |
| 142 | ATF4 |  |  | 4850 | -0.027 | -0.3873 | No |
| 143 | EZH2 |  |  | 4858 | -0.028 | -0.3879 | No |
| 144 | ZNF217 |  |  | 4859 | -0.028 | -0.3877 | No |
| 145 | WBP4 |  |  | 4908 | -0.029 | -0.3931 | No |
| 146 | ARRDC3 |  |  | 4938 | -0.031 | -0.3963 | No |
| 147 | DDX39A |  |  | 4948 | -0.031 | -0.3971 | No |
| 148 | KIF18A |  |  | 4962 | -0.032 | -0.3983 | No |
| 149 | EIF1AD |  |  | 4966 | -0.032 | -0.3984 | No |
| 150 | SP3 |  |  | 4985 | -0.032 | -0.4003 | No |
| 151 | PPIL4 |  |  | 5022 | -0.034 | -0.4042 | No |
| 152 | ZDHHC2 |  |  | 5023 | -0.034 | -0.4040 | No |
| 153 | HAUS8 |  |  | 5039 | -0.034 | -0.4054 | No |
| 154 | TYW3 |  |  | 5086 | -0.037 | -0.4105 | No |
| 155 | MED30 |  |  | 5094 | -0.037 | -0.4111 | No |
| 156 | CCNB1 |  |  | 5111 | -0.037 | -0.4126 | No |
| 157 | SPAG5 |  |  | 5225 | -0.041 | -0.4255 | No |
| 158 | LEF1 |  |  | 5255 | -0.042 | -0.4286 | No |
| 159 | ECT2 |  |  | 5264 | -0.043 | -0.4292 | No |
| 160 | TP53 |  |  | 5275 | -0.043 | -0.4300 | No |
| 161 | NCAPG2 |  |  | 5309 | -0.045 | -0.4335 | No |
| 162 | SPC25 |  |  | 5312 | -0.045 | -0.4334 | No |
| 163 | PPP1R15A |  |  | 5321 | -0.045 | -0.4340 | No |
| 164 | DDIT3 |  |  | 5335 | -0.046 | -0.4351 | No |
| 165 | UHRF1 |  |  | 5337 | -0.046 | -0.4349 | No |
| 166 | CCNB2 |  |  | 5382 | -0.048 | -0.4397 | No |
| 167 | NUSAP1 |  |  | 5389 | -0.048 | -0.4400 | No |
| 168 | TCF3 |  |  | 5402 | -0.048 | -0.4410 | No |
| 169 | RSRC2 |  |  | 5447 | -0.050 | -0.4457 | No |
| 170 | ERRFI1 |  |  | 5451 | -0.050 | -0.4457 | No |
| 171 | PHLPP1 |  |  | 5506 | -0.053 | -0.4516 | No |
| 172 | ZFAND2A |  |  | 5562 | -0.056 | -0.4576 | No |
| 173 | IFT74 |  |  | 5565 | -0.056 | -0.4574 | No |
| 174 | YAP1 |  |  | 5575 | -0.056 | -0.4580 | No |
| 175 | TTK |  |  | 5604 | -0.058 | -0.4608 | No |
| 176 | NEK4 |  |  | 5654 | -0.060 | -0.4661 | No |
| 177 | ZCCHC3 |  |  | 5656 | -0.060 | -0.4657 | No |
| 178 | CCNA2 |  |  | 5671 | -0.061 | -0.4669 | No |
| 179 | TIMELESS |  |  | 5717 | -0.063 | -0.4716 | No |
| 180 | TOP2A |  |  | 5722 | -0.063 | -0.4716 | No |
| 181 | CDCA3 |  |  | 5726 | -0.063 | -0.4715 | No |
| 182 | KIF22 |  |  | 5758 | -0.064 | -0.4746 | No |
| 183 | NOP58 |  |  | 5766 | -0.064 | -0.4749 | No |
| 184 | EAF1 |  |  | 5786 | -0.065 | -0.4766 | No |
| 185 | KIF11 |  |  | 5795 | -0.065 | -0.4770 | No |
| 186 | DHX40 |  |  | 5864 | -0.068 | -0.4844 | No |
| 187 | MRPL50 |  |  | 5877 | -0.069 | -0.4853 | No |
| 188 | CHMP1B |  |  | 5886 | -0.069 | -0.4857 | No |
| 189 | RACGAP1 |  |  | 5902 | -0.070 | -0.4869 | No |
| 190 | KNSTRN |  |  | 5923 | -0.070 | -0.4887 | No |
| 191 | TFAP2C |  |  | 6012 | -0.075 | -0.4984 | No |
| 192 | PPM1D |  |  | 6025 | -0.076 | -0.4992 | No |
| 193 | SFRP1 |  |  | 6042 | -0.076 | -0.5005 | No |
| 194 | ATP6V0E1 |  |  | 6138 | -0.082 | -0.5109 | No |
| 195 | G3BP1 |  |  | 6152 | -0.082 | -0.5118 | No |
| 196 | ADNP |  |  | 6175 | -0.083 | -0.5137 | No |
| 197 | MKI67 |  |  | 6211 | -0.086 | -0.5171 | No |
| 198 | KCTD6 |  |  | 6217 | -0.086 | -0.5170 | No |
| 199 | PDCD4 |  |  | 6228 | -0.086 | -0.5175 | No |
| 200 | PSMD14 |  |  | 6275 | -0.089 | -0.5222 | No |
| 201 | RRM2 |  |  | 6308 | -0.091 | -0.5252 | No |
| 202 | RPF2 |  |  | 6326 | -0.091 | -0.5265 | No |
| 203 | CKAP2 |  |  | 6372 | -0.094 | -0.5310 | No |
| 204 | TLK1 |  |  | 6386 | -0.094 | -0.5318 | No |
| 205 | ARHGAP11A |  |  | 6423 | -0.097 | -0.5352 | No |
| 206 | CDC42EP4 |  |  | 6451 | -0.099 | -0.5376 | No |
| 207 | ZWINT |  |  | 6453 | -0.099 | -0.5369 | No |
| 208 | TJP1 |  |  | 6500 | -0.101 | -0.5415 | No |
| 209 | SMC4 |  |  | 6517 | -0.102 | -0.5426 | No |
| 210 | KIF4A |  |  | 6529 | -0.103 | -0.5431 | No |
| 211 | THUMPD1 |  |  | 6552 | -0.105 | -0.5448 | No |
| 212 | DBF4 |  |  | 6572 | -0.106 | -0.5462 | No |
| 213 | HSPA1B |  |  | 6593 | -0.108 | -0.5477 | No |
| 214 | MAD2L1 |  |  | 6600 | -0.108 | -0.5475 | No |
| 215 | HSP90AB1 |  |  | 6611 | -0.109 | -0.5478 | No |
| 216 | RYBP |  |  | 6649 | -0.111 | -0.5513 | No |
| 217 | BET1 |  |  | 6654 | -0.111 | -0.5509 | No |
| 218 | ANP32E |  |  | 6655 | -0.111 | -0.5500 | No |
| 219 | GPN1 |  |  | 6673 | -0.112 | -0.5511 | No |
| 220 | MIER1 |  |  | 6728 | -0.116 | -0.5565 | No |
| 221 | TIPARP |  |  | 6731 | -0.116 | -0.5558 | No |
| 222 | KIF23 |  |  | 6764 | -0.118 | -0.5586 | No |
| 223 | ANLN |  |  | 6829 | -0.123 | -0.5651 | No |
| 224 | HSPH1 |  |  | 6836 | -0.124 | -0.5649 | No |
| 225 | NFYB |  |  | 6899 | -0.129 | -0.5711 | No |
| 226 | PBDC1 |  |  | 6921 | -0.131 | -0.5725 | No |
| 227 | AHSA1 |  |  | 6923 | -0.131 | -0.5716 | No |
| 228 | TGIF1 |  |  | 6926 | -0.131 | -0.5708 | No |
| 229 | ZEB1 |  |  | 6944 | -0.132 | -0.5717 | No |
| 230 | FXR1 |  |  | 6948 | -0.133 | -0.5711 | No |
| 231 | GSK3B |  |  | 6951 | -0.133 | -0.5702 | No |
| 232 | HSP90AA1 |  |  | 6962 | -0.134 | -0.5704 | No |
| 233 | MND1 |  |  | 6972 | -0.135 | -0.5703 | No |
| 234 | PBK |  |  | 7010 | -0.138 | -0.5736 | No |
| 235 | TBL1X |  |  | 7026 | -0.139 | -0.5742 | No |
| 236 | CCDC59 |  |  | 7079 | -0.144 | -0.5792 | No |
| 237 | CASP3 |  |  | 7082 | -0.144 | -0.5783 | No |
| 238 | RBBP8 |  |  | 7099 | -0.145 | -0.5790 | No |
| 239 | SVIP |  |  | 7114 | -0.146 | -0.5795 | No |
| 240 | CENPW |  |  | 7116 | -0.147 | -0.5784 | No |
| 241 | RPL22L1 |  |  | 7150 | -0.150 | -0.5811 | No |
| 242 | USP39 |  |  | 7176 | -0.153 | -0.5828 | No |
| 243 | BTBD1 |  |  | 7177 | -0.154 | -0.5816 | No |
| 244 | GMNN |  |  | 7224 | -0.158 | -0.5858 | No |
| 245 | PIN4 |  |  | 7284 | -0.166 | -0.5913 | No |
| 246 | IRF2BP2 |  |  | 7287 | -0.166 | -0.5903 | No |
| 247 | EIF3J |  |  | 7306 | -0.167 | -0.5911 | No |
| 248 | ZFP36L2 |  |  | 7317 | -0.169 | -0.5909 | No |
| 249 | HEY1 |  |  | 7370 | -0.177 | -0.5956 | No |
| 250 | SNRPA1 |  |  | 7376 | -0.178 | -0.5948 | No |
| 251 | USP1 |  |  | 7398 | -0.181 | -0.5958 | No |
| 252 | PSIP1 |  |  | 7460 | -0.187 | -0.6014 | No |
| 253 | ZFP36L1 |  |  | 7466 | -0.188 | -0.6005 | No |
| 254 | OSER1 |  |  | 7496 | -0.190 | -0.6024 | No |
| 255 | CENPN |  |  | 7528 | -0.194 | -0.6045 | No |
| 256 | CMBL |  |  | 7534 | -0.195 | -0.6036 | No |
| 257 | TPX2 |  |  | 7549 | -0.197 | -0.6037 | No |
| 258 | MCM3 |  |  | 7552 | -0.198 | -0.6023 | No |
| 259 | UBE2T |  |  | 7561 | -0.199 | -0.6017 | No |
| 260 | BIRC5 |  |  | 7596 | -0.204 | -0.6041 | No |
| 261 | SALL1 |  |  | 7597 | -0.204 | -0.6025 | No |
| 262 | BTG3 |  |  | 7602 | -0.204 | -0.6013 | No |
| 263 | CAPZA1 |  |  | 7607 | -0.205 | -0.6002 | No |
| 264 | WTAP |  |  | 7646 | -0.210 | -0.6030 | No |
| 265 | TOMM5 |  |  | 7655 | -0.211 | -0.6022 | No |
| 266 | PPP4R2 |  |  | 7660 | -0.212 | -0.6010 | No |
| 267 | MAP1B |  |  | 7666 | -0.213 | -0.5999 | No |
| 268 | ETF1 |  |  | 7748 | -0.225 | -0.6076 | No |
| 269 | MRPL47 |  |  | 7759 | -0.226 | -0.6070 | No |
| 270 | TAF7 |  |  | 7793 | -0.233 | -0.6090 | No |
| 271 | TAF9 |  |  | 7817 | -0.238 | -0.6098 | No |
| 272 | FDFT1 |  |  | 7852 | -0.244 | -0.6119 | Yes |
| 273 | CCDC25 |  |  | 7855 | -0.244 | -0.6102 | Yes |
| 274 | PNRC1 |  |  | 7867 | -0.247 | -0.6095 | Yes |
| 275 | COPS8 |  |  | 7869 | -0.248 | -0.6077 | Yes |
| 276 | CENPU |  |  | 7871 | -0.248 | -0.6059 | Yes |
| 277 | SSB |  |  | 7906 | -0.257 | -0.6078 | Yes |
| 278 | SLIRP |  |  | 7914 | -0.258 | -0.6066 | Yes |
| 279 | RSL24D1 |  |  | 7919 | -0.259 | -0.6050 | Yes |
| 280 | EIF1B |  |  | 7931 | -0.262 | -0.6042 | Yes |
| 281 | TWISTNB |  |  | 7937 | -0.263 | -0.6027 | Yes |
| 282 | CKS2 |  |  | 7972 | -0.270 | -0.6046 | Yes |
| 283 | HMGB2 |  |  | 7973 | -0.271 | -0.6025 | Yes |
| 284 | HIST1H1C |  |  | 7978 | -0.271 | -0.6008 | Yes |
| 285 | FAM162A |  |  | 7983 | -0.272 | -0.5991 | Yes |
| 286 | PAPOLA |  |  | 7986 | -0.272 | -0.5972 | Yes |
| 287 | PSMA3 |  |  | 8006 | -0.277 | -0.5972 | Yes |
| 288 | TPM1 |  |  | 8012 | -0.278 | -0.5956 | Yes |
| 289 | ACAA2 |  |  | 8017 | -0.279 | -0.5939 | Yes |
| 290 | PPP1CC |  |  | 8035 | -0.282 | -0.5937 | Yes |
| 291 | TMSB15A |  |  | 8046 | -0.284 | -0.5926 | Yes |
| 292 | TRIAP1 |  |  | 8060 | -0.288 | -0.5918 | Yes |
| 293 | CTNNAL1 |  |  | 8076 | -0.292 | -0.5913 | Yes |
| 294 | CDKN3 |  |  | 8079 | -0.293 | -0.5892 | Yes |
| 295 | PCNA |  |  | 8082 | -0.293 | -0.5871 | Yes |
| 296 | PSMA1 |  |  | 8109 | -0.299 | -0.5878 | Yes |
| 297 | DUT |  |  | 8118 | -0.300 | -0.5864 | Yes |
| 298 | RPS27L |  |  | 8126 | -0.302 | -0.5848 | Yes |
| 299 | TCP1 |  |  | 8137 | -0.306 | -0.5836 | Yes |
| 300 | HIST1H1A |  |  | 8148 | -0.309 | -0.5823 | Yes |
| 301 | HIST1H1E |  |  | 8176 | -0.320 | -0.5829 | Yes |
| 302 | SRSF3 |  |  | 8191 | -0.323 | -0.5820 | Yes |
| 303 | CCT4 |  |  | 8211 | -0.329 | -0.5816 | Yes |
| 304 | KIF5B |  |  | 8235 | -0.334 | -0.5817 | Yes |
| 305 | CKS1B |  |  | 8241 | -0.336 | -0.5796 | Yes |
| 306 | RBM8A |  |  | 8253 | -0.340 | -0.5782 | Yes |
| 307 | BNIP3 |  |  | 8255 | -0.341 | -0.5757 | Yes |
| 308 | LDHA |  |  | 8263 | -0.343 | -0.5738 | Yes |
| 309 | PPA1 |  |  | 8265 | -0.344 | -0.5712 | Yes |
| 310 | UBE2C |  |  | 8278 | -0.348 | -0.5698 | Yes |
| 311 | ANAPC16 |  |  | 8286 | -0.350 | -0.5679 | Yes |
| 312 | CSNK2B |  |  | 8298 | -0.354 | -0.5664 | Yes |
| 313 | SOX9 |  |  | 8327 | -0.361 | -0.5668 | Yes |
| 314 | GLO1 |  |  | 8338 | -0.364 | -0.5651 | Yes |
| 315 | DPY30 |  |  | 8360 | -0.374 | -0.5646 | Yes |
| 316 | LDHB |  |  | 8372 | -0.379 | -0.5629 | Yes |
| 317 | NAP1L1 |  |  | 8404 | -0.389 | -0.5635 | Yes |
| 318 | NOL7 |  |  | 8405 | -0.389 | -0.5604 | Yes |
| 319 | PUF60 |  |  | 8407 | -0.390 | -0.5575 | Yes |
| 320 | HSPD1 |  |  | 8418 | -0.393 | -0.5555 | Yes |
| 321 | HMGB3 |  |  | 8427 | -0.399 | -0.5533 | Yes |
| 322 | SNRPE |  |  | 8435 | -0.401 | -0.5510 | Yes |
| 323 | HIST1H1B |  |  | 8437 | -0.402 | -0.5479 | Yes |
| 324 | PTTG1 |  |  | 8446 | -0.406 | -0.5456 | Yes |
| 325 | CKB |  |  | 8448 | -0.407 | -0.5425 | Yes |
| 326 | EEF1D |  |  | 8455 | -0.412 | -0.5400 | Yes |
| 327 | NDUFB1 |  |  | 8462 | -0.415 | -0.5374 | Yes |
| 328 | H1FX |  |  | 8466 | -0.417 | -0.5345 | Yes |
| 329 | CACYBP |  |  | 8486 | -0.428 | -0.5333 | Yes |
| 330 | PEA15 |  |  | 8509 | -0.439 | -0.5324 | Yes |
| 331 | HIST1H1D |  |  | 8520 | -0.443 | -0.5301 | Yes |
| 332 | SNRPD1 |  |  | 8537 | -0.451 | -0.5284 | Yes |
| 333 | PSMB7 |  |  | 8541 | -0.452 | -0.5252 | Yes |
| 334 | RAN |  |  | 8555 | -0.463 | -0.5231 | Yes |
| 335 | PSMB3 |  |  | 8576 | -0.472 | -0.5217 | Yes |
| 336 | SNRPG |  |  | 8578 | -0.473 | -0.5181 | Yes |
| 337 | HSPB1 |  |  | 8586 | -0.478 | -0.5151 | Yes |
| 338 | PTGES3 |  |  | 8587 | -0.479 | -0.5114 | Yes |
| 339 | TYMS |  |  | 8599 | -0.488 | -0.5088 | Yes |
| 340 | YBX3 |  |  | 8604 | -0.493 | -0.5054 | Yes |
| 341 | HMGN2 |  |  | 8608 | -0.496 | -0.5018 | Yes |
| 342 | YWHAE |  |  | 8629 | -0.512 | -0.5001 | Yes |
| 343 | SLC25A3 |  |  | 8637 | -0.517 | -0.4968 | Yes |
| 344 | CYCS |  |  | 8640 | -0.519 | -0.4930 | Yes |
| 345 | HSPE1 |  |  | 8650 | -0.524 | -0.4899 | Yes |
| 346 | HMGB1 |  |  | 8653 | -0.527 | -0.4860 | Yes |
| 347 | SIVA1 |  |  | 8654 | -0.529 | -0.4818 | Yes |
| 348 | VIM |  |  | 8663 | -0.535 | -0.4785 | Yes |
| 349 | PRDX6 |  |  | 8673 | -0.540 | -0.4753 | Yes |
| 350 | HMGA1 |  |  | 8675 | -0.543 | -0.4711 | Yes |
| 351 | SNRPB |  |  | 8683 | -0.547 | -0.4676 | Yes |
| 352 | HMGN3 |  |  | 8684 | -0.547 | -0.4633 | Yes |
| 353 | NDUFA6 |  |  | 8687 | -0.550 | -0.4592 | Yes |
| 354 | SCAND1 |  |  | 8689 | -0.550 | -0.4550 | Yes |
| 355 | RHEB |  |  | 8694 | -0.554 | -0.4511 | Yes |
| 356 | PGAM1 |  |  | 8698 | -0.558 | -0.4471 | Yes |
| 357 | SRP14 |  |  | 8701 | -0.565 | -0.4428 | Yes |
| 358 | NPM1 |  |  | 8706 | -0.566 | -0.4388 | Yes |
| 359 | RPL13A |  |  | 8714 | -0.578 | -0.4351 | Yes |
| 360 | POLR2L |  |  | 8715 | -0.578 | -0.4305 | Yes |
| 361 | UQCRFS1 |  |  | 8720 | -0.582 | -0.4264 | Yes |
| 362 | ENO1 |  |  | 8734 | -0.593 | -0.4233 | Yes |
| 363 | UBB |  |  | 8745 | -0.602 | -0.4197 | Yes |
| 364 | CNN3 |  |  | 8752 | -0.613 | -0.4155 | Yes |
| 365 | RPS21 |  |  | 8761 | -0.620 | -0.4116 | Yes |
| 366 | RPL10A |  |  | 8765 | -0.623 | -0.4070 | Yes |
| 367 | DEK |  |  | 8770 | -0.627 | -0.4025 | Yes |
| 368 | H2AFZ |  |  | 8774 | -0.630 | -0.3979 | Yes |
| 369 | RPL5 |  |  | 8780 | -0.635 | -0.3935 | Yes |
| 370 | RPL23 |  |  | 8781 | -0.636 | -0.3885 | Yes |
| 371 | RPL14 |  |  | 8787 | -0.642 | -0.3840 | Yes |
| 372 | RPS7 |  |  | 8791 | -0.644 | -0.3793 | Yes |
| 373 | RPL7 |  |  | 8792 | -0.644 | -0.3742 | Yes |
| 374 | GSTP1 |  |  | 8795 | -0.647 | -0.3693 | Yes |
| 375 | POLR2J |  |  | 8798 | -0.648 | -0.3644 | Yes |
| 376 | RPL21 |  |  | 8802 | -0.655 | -0.3596 | Yes |
| 377 | PHLDA1 |  |  | 8805 | -0.655 | -0.3547 | Yes |
| 378 | RPL37 |  |  | 8808 | -0.656 | -0.3498 | Yes |
| 379 | RPS24 |  |  | 8832 | -0.688 | -0.3470 | Yes |
| 380 | ODC1 |  |  | 8839 | -0.697 | -0.3422 | Yes |
| 381 | H3F3B |  |  | 8846 | -0.705 | -0.3374 | Yes |
| 382 | HIST1H4C |  |  | 8850 | -0.710 | -0.3321 | Yes |
| 383 | LSM4 |  |  | 8853 | -0.712 | -0.3267 | Yes |
| 384 | RPL24 |  |  | 8856 | -0.715 | -0.3213 | Yes |
| 385 | RPL15 |  |  | 8863 | -0.724 | -0.3163 | Yes |
| 386 | GNG5 |  |  | 8866 | -0.728 | -0.3108 | Yes |
| 387 | RPS27A |  |  | 8875 | -0.742 | -0.3059 | Yes |
| 388 | RPL35 |  |  | 8892 | -0.763 | -0.3018 | Yes |
| 389 | RPL39 |  |  | 8893 | -0.763 | -0.2957 | Yes |
| 390 | RPLP0 |  |  | 8904 | -0.777 | -0.2908 | Yes |
| 391 | RPS28 |  |  | 8906 | -0.778 | -0.2847 | Yes |
| 392 | RPL18 |  |  | 8907 | -0.780 | -0.2786 | Yes |
| 393 | RPS25 |  |  | 8909 | -0.787 | -0.2725 | Yes |
| 394 | PPIA |  |  | 8913 | -0.797 | -0.2666 | Yes |
| 395 | GAPDH |  |  | 8915 | -0.803 | -0.2604 | Yes |
| 396 | RPL9 |  |  | 8918 | -0.813 | -0.2542 | Yes |
| 397 | SERF2 |  |  | 8920 | -0.816 | -0.2479 | Yes |
| 398 | RPS18 |  |  | 8926 | -0.829 | -0.2419 | Yes |
| 399 | RPS26 |  |  | 8928 | -0.835 | -0.2354 | Yes |
| 400 | RPS6 |  |  | 8929 | -0.837 | -0.2288 | Yes |
| 401 | RPLP2 |  |  | 8932 | -0.844 | -0.2224 | Yes |
| 402 | NACA |  |  | 8933 | -0.844 | -0.2157 | Yes |
| 403 | RPL23A |  |  | 8934 | -0.845 | -0.2091 | Yes |
| 404 | RPS3 |  |  | 8936 | -0.850 | -0.2025 | Yes |
| 405 | RPL30 |  |  | 8937 | -0.850 | -0.1958 | Yes |
| 406 | MYL6 |  |  | 8938 | -0.851 | -0.1891 | Yes |
| 407 | TPI1 |  |  | 8939 | -0.852 | -0.1824 | Yes |
| 408 | RPS23 |  |  | 8940 | -0.854 | -0.1756 | Yes |
| 409 | RPS19 |  |  | 8941 | -0.855 | -0.1689 | Yes |
| 410 | RPL6 |  |  | 8942 | -0.856 | -0.1621 | Yes |
| 411 | RPL13 |  |  | 8944 | -0.862 | -0.1555 | Yes |
| 412 | RPL12 |  |  | 8945 | -0.864 | -0.1486 | Yes |
| 413 | RPS14 |  |  | 8949 | -0.877 | -0.1421 | Yes |
| 414 | RPL32 |  |  | 8953 | -0.891 | -0.1354 | Yes |
| 415 | RPS3A |  |  | 8957 | -0.906 | -0.1286 | Yes |
| 416 | EEF1A1 |  |  | 8958 | -0.915 | -0.1214 | Yes |
| 417 | PKM |  |  | 8959 | -0.915 | -0.1142 | Yes |
| 418 | RPS2 |  |  | 8960 | -0.923 | -0.1069 | Yes |
| 419 | HINT1 |  |  | 8961 | -0.923 | -0.0996 | Yes |
| 420 | RPL19 |  |  | 8962 | -0.925 | -0.0923 | Yes |
| 421 | RPL7A |  |  | 8963 | -0.925 | -0.0850 | Yes |
| 422 | RPL29 |  |  | 8964 | -0.930 | -0.0777 | Yes |
| 423 | GPX4 |  |  | 8966 | -0.946 | -0.0703 | Yes |
| 424 | TPT1 |  |  | 8967 | -0.951 | -0.0628 | Yes |
| 425 | RPLP1 |  |  | 8968 | -0.958 | -0.0553 | Yes |
| 426 | RPL18A |  |  | 8969 | -0.964 | -0.0477 | Yes |
| 427 | RPS15 |  |  | 8970 | -0.965 | -0.0401 | Yes |
| 428 | RPL8 |  |  | 8971 | -0.992 | -0.0322 | Yes |
| 429 | RPL41 |  |  | 8974 | -1.013 | -0.0245 | Yes |
| 430 | RPS12 |  |  | 8975 | -1.034 | -0.0163 | Yes |
| 431 | RPS8 |  |  | 8976 | -1.044 | -0.0081 | Yes |
| 432 | FTH1 |  |  | 8981 | -1.131 | 0.0004 | Yes |
Table: GSEA details [plain text format]

  

Fig 2: RICHARDS\_NOWAKOWSKI\_SCIENCE\_2017\_RG\_EARLY\_UPREG: Random ES distribution      
 Gene set null distribution of ES for **RICHARDS\_NOWAKOWSKI\_SCIENCE\_2017\_RG\_EARLY\_UPREG**
